# HEADPLAY Personal Cinema System Facilitates Intravenous Cannulation in Children: A Randomized Controlled Trial

**DOI:** 10.1155/2013/849469

**Published:** 2013-06-11

**Authors:** Evangeline Lim, Teddy Fabila, Thong Sze Ying, Josephine Tan

**Affiliations:** ^1^Department of Paediatric Anaesthesia, KK Women's and Children's Hospital, Singapore; ^2^Department of Anaesthesiology, Singapore General Hospital, Singapore

## Abstract

HEADPLAY personal cinema system (PCS) is a portable visual headset/visor through which movie clips may be viewed. We studied the use of HEADPLAY PCS as a distraction tool in facilitating intravenous cannulation in children undergoing anaesthesia. 60 children were enrolled into the study and randomized into 2 groups. EMLA local anaesthetic cream was used to reduce the pain associated with intravenous cannulation. Children in group 1 wore the HEADPLAY visor whereas children in group 2 were subject to conventional distraction therapy. Children were asked to rate their anxiety, pain, and satisfaction scores after intravenous cannulation. Periprocedural anxiety was also determined using the modified Yale Preoperative Anxiety Scale (mYPAS). There were no statistically significant differences in terms of pain and anxiety scores between the 2 groups. Although the satisfaction score of the children in the HEADPLAY PCS group was marginally higher compared to the conventional group, this did not hit statistical significance. 86.6% of children in group 1 reported that they would want to use the visor again for their next intravenous cannulation. We conclude that HEADPLAY PCS is a distraction tool that is acceptable to most children and can contribute towards satisfaction of the intravenous cannulation process in children.

## 1. Introduction

Intravenous cannulation without inducing anxiety or pain in the awake child is desirable but not always attainable even with topical anaesthetics applied. Even if the topical anaesthetic is completely effective, it may not necessarily remove anticipated anxiety associated with what is normally deemed a painful procedure. For children undergoing surgery, anaesthetic induction appears to be the greatest stressor in the perioperative period [1]. There are numerous factors that contribute towards this, including the child's age and personality [2], the fear of separation from parents, the fear of pain or surgery itself, and even parental anxiety [2, 3]. While mask induction may seem the less traumatic choice, it is well observed amongst paediatric anaesthetists that mask phobia exists in as much as needle phobia does [4]. In our institution, children are not routinely premedicated before anaesthesia, and intravenous cannulation is performed after EMLA cream is applied for 60 min, allowing sufficient time for it to have an effect. Age appropriate distraction therapy tools are usually employed during this process. We sought to study if HEADPLAY PCS was as effective as conventional therapy used at our institution for facilitating the insertion of an intravenous cannula in children undergoing intravenous induction. 

**Figure 1:**
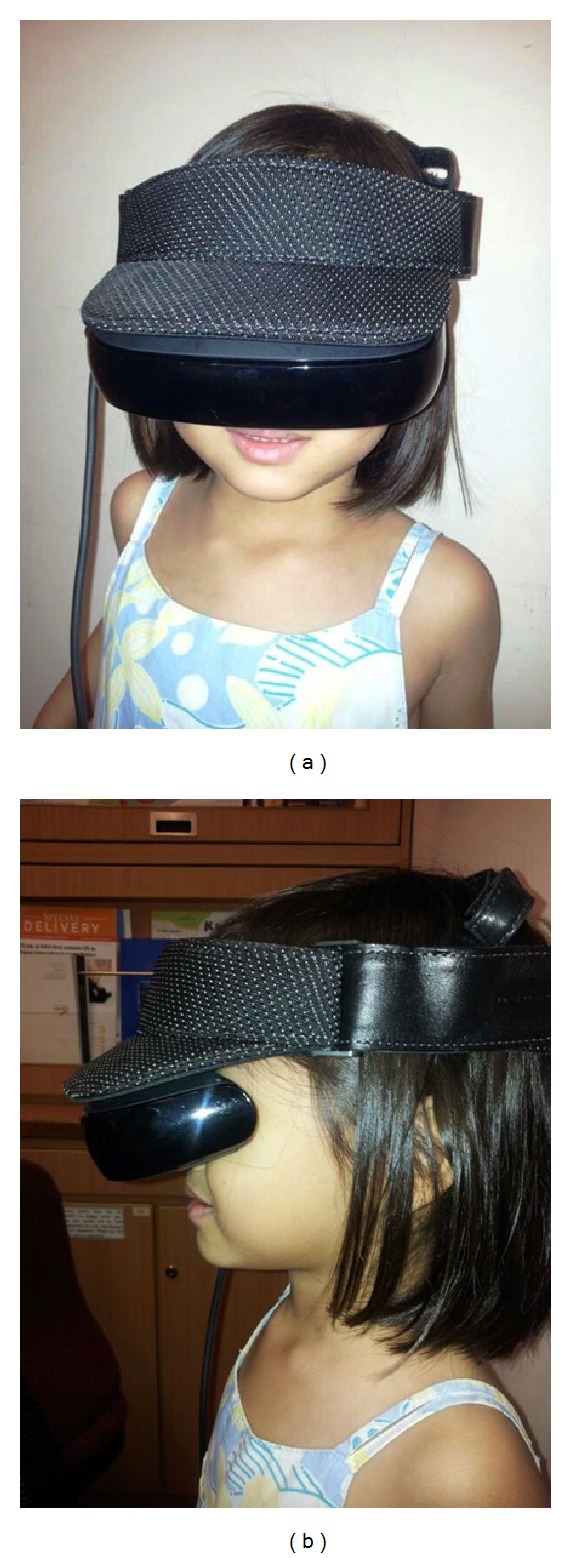
Child wearing HEADPLAY visor.

HEADPLAY PCS is a portable, visual headset and multimedia center that is capable of delivering high resolution, cinematic viewing in an immersive environment (Figure 1). It may be used for gaming, movie watching, and internet use. It has the potential to be a powerful tool in alleviating preoperative anxiety for medical procedures such as intravenous cannulation. For this study, we used it for movie viewing. 

## 2. Methods

Institutional review board approval (CIRB ref: 2010/526/D) was obtained for this study. This was a prospective randomised controlled trial involving 60 children. The inclusion criteria included any ASA I-III child between the age of 5 and 15 years who was able to understand what the study entailed. EMLA had to be applied for at least 1 h before the child was approached for recruitment. It was also necessary for the child's head to be able to accommodate the HEADPLAY visor. The child should also have not been administered with any painkiller or premedication within the previous 24 hours. Exclusion criteria included children who were allergic to EMLA, ASA IV patients, those with an indwelling cannula in place, and those with heads that could not accommodate the visor. Recruitment was conducted entirely by anaesthetists who were the investigators of this study. To aid recruitment, all children were allowed to use the HEADPLAY PCS device for a minute in the holding area outside the operating theatre (Figure 1), but it was made clear to the child and parents that consequent to randomization, the child may not have the opportunity to use the device during the first intravenous cannulation attempt. The conventional method of distraction therapy used in our institution was also explained to both the parent and child to facilitate them making an informed decision about whether to participate in the study.

The 60 children were randomized into 2 groups of 30 each using a computer-generated random table. Group 1 used HEADPLAY PCS during the intravenous cannulation. Group 2 had conventional methods of distraction used during intravenous cannula insertion. This would include verbal distraction, bubble play, or choosing stickers.

After preoperative screening, all eligible cases for the study were approached and parental consent was obtained. Assent was obtained from the child. The child's anxiety and pain scores were assessed in the holding area by a trained anaesthetic nurse using the modified Yale Preoperative Anxiety Scale (mYPAS). In both groups, the child was also instructed on the use of the Wong Baker Faces rating scale and did a self-reporting score of anxiety and baseline pain, with 0 representing no pain/anxiety and 10 representing the worst possible pain/most anxious. The parent was asked to assess the degree of anxiety of his/her own child with scores ranging from 1 (not anxious/scared), 2 (a little anxious/scared), 3 (anxious/scared), 4 (very anxious/scared), and 5 (very, very anxious/scared).

**Figure 2:**
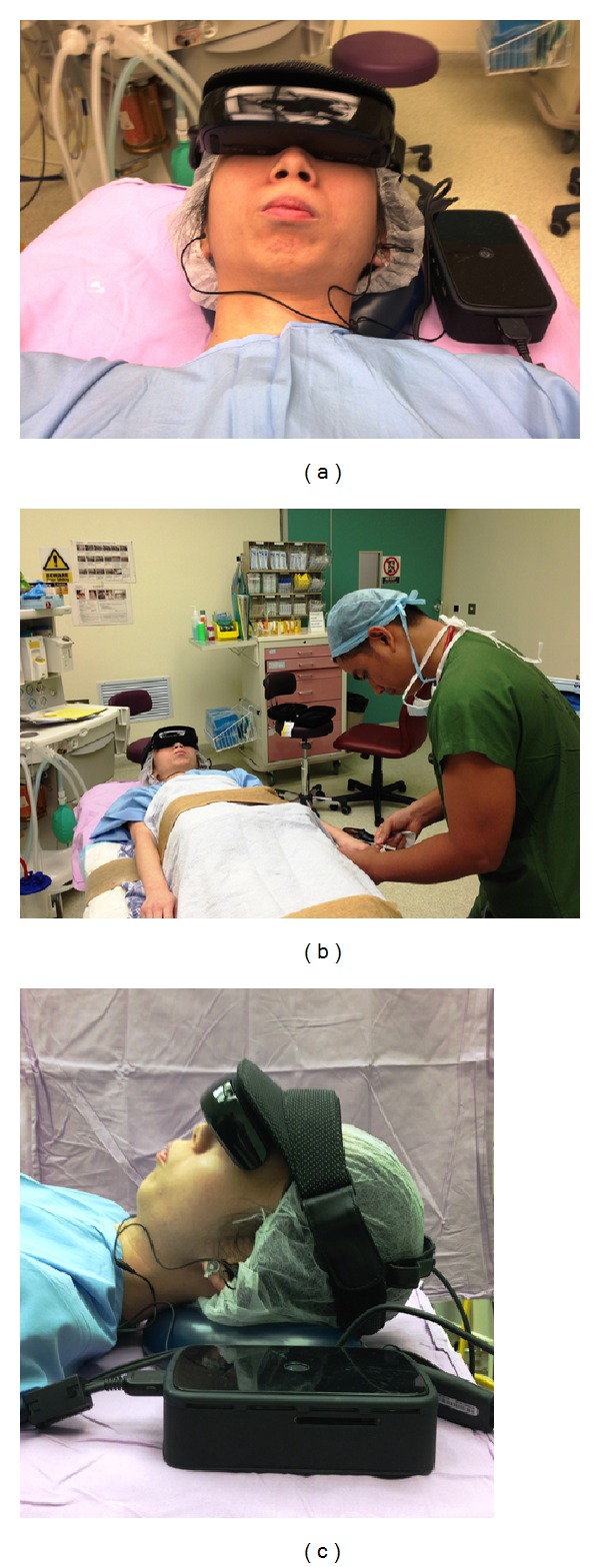
The use of HEADPLAY PCS device during intravenous cannula insertion.

The child was then taken into the operating theatre (OT). In the OT, the child was asked for his/her anxiety scores from 0 to 10. Concurrently, his anxiety was assessed by the same anaesthetic nurse using mYPAS. Depending on the group to which the child had been randomized, standard distraction therapy or HEADPLAY PCS was administered to the child before intravenous cannulation (Figure 2). Children in group 1 had the HEADPLAY PCS device on for 2 minutes before the cannulation attempt was made.

The intravenous cannula was inserted by the investigator who is an experienced anaesthetist. The child was assessed with the mYPAS score during the cannulation process by the same anaesthetic nurse who had assessed the child before. The child was asked to report on pain and satisfaction with the overall process after the first intravenous cannulation attempt was made, whether it was successful or not. The child was also assessed by the nurse with regard to cooperation during the cannulation attempt. 

If the first attempt at cannulation failed, the child was not assessed further for pain and anxiety although further attempts at cannulation may have been made with a distraction technique chosen by the child.

If the child rejected the HEADPLAY PCS device half way through, this was regarded as a failure under the study group but assessment of the child would still continue with the alternative standard distraction therapy. 

Children under the HEADPLAY PCS device group were further asked if they liked the device, whether they found it comfortable, and if they would like to use it again for future intravenous cannulation. Based on a previous pilot study of 20 patients, in order to detect a 25% difference in satisfaction scores between the HEADPLAY PCS device group and the control group, a sample size of 30 was required in each group. All data was analyzed using SPSS 15.0 software. Parametric patient demographics such as age and satisfaction score were analyzed using *t*-test. All nonparametric data such as gender, ethnicity, ASA status, and previous intravenous cannula insertion were analyzed using Chi-square test/Fisher's exact test as appropriate. Anxiety scores and pain scores within groups were analyzed using independent sample Wilcoxon rank-sum (Mann-Whitney *U*) test. Spearman correlation was used to analyze the association between pain and satisfaction rate. Data is presented as mean ± standard deviation (SD). A *P* value of less than 0.05 was considered statistically significant. 

## 3. Results

All patients complied with their allocated groups. 

### 3.1. Subject Demographics

Patient demographics are summarized in Table 1. There were no statistically significant differences between the 2 groups. 29 children in each group had one parent with them during the induction. The remaining one in each group elected to enter the operating theatre alone. The majority of children recruited were Chinese or Malay boys with no previous intravenous cannulation experience. Of those who had previous intravenous cannulation, none of them had cried during the cannulation process. 

### 3.2. Assessment of Anxiety

Table 2 summarizes the anxiety scores of children obtained from self-assessment, parental observation and using the mYPAS score. There was no statistically significant difference in terms of self-assessment anxiety scores of children in the conventional technique group and the HEADPLAY PCS group. When the children's anxiety levels were observed by the trained personnel using the mYPAS, even though it appeared that there was a trend towards increasing anxiety as the child progressed from the holding room to the operating theatre and intravenous cannulation, both groups were comparable in terms of their anxiety level at each stage. The increase in anxiety levels between each location for each child was also not statistically significant. Parental assessment of the child's anxiety level in the holding room was similar for both groups. 

### 3.3. Pain Assessment and Satisfaction Scores

Pain scores at the holding area were negligible for both groups. The HEADPLAY PCS group had a slightly higher pain score related to intravenous cannulation but also a trend towards higher satisfaction scores than the conventional technique group (Table 3). These differences did not reach statistical significance. In both groups, there was a negative correlation between pain and satisfaction (Spearman's correlation coefficient −0.46 in group 1 versus −0.66 in group 2). The HEADPLAY PCS device was fairly well accepted by the children (Table 4). There was not always a correlation between liking the device and wanting to use it again. One child who liked the device and found it comfortable did not want to use it again. Conversely, two children who did not like the device wanted to use it again for their next intravenous cannulation. Three children who did not like the device and did not want to use it again found the device uncomfortable; of these, two had significant pain scores associated with intravenous cannulation of more than 4.

## 4. Discussion

Medical procedures and surgery are sources of pain and anxiety for patients. In children undergoing surgery, preoperative anxiety can translate into negative physiological and behavioural responses that persist into the postoperative period such as crying, sleep disturbance, separation anxiety, and increased postoperative pain [5, 6]. The degree of preoperative anxiety can depend on the age and personality of the child [2] and the environmental set-up of the holding room and the operating theatre. Besides building a good rapport with the child and parents/caregiver, both pharmacologic and nonpharmacologic options may be effective in relieving anxiety in a child [7]. Pharmacologic interventions given at appropriate doses will alleviate anxiety, but not without the risk of side/adverse effects [8]. Nonpharmacologic interventions that have been employed to reduce preoperative anxiety and needle-related procedural pain and distress in children include music, acupuncture/acupressure [9], clowns [10], parental presence/distraction [11], hand-held games/electronic devices, smart phones [12, 13], toys, bubbles, and cartoons [14]. Systematic reviews of the use of psychological interventions in children undergoing needle-related procedures like routine childhood immunization suggest that breathing exercises, child-directed distraction, nurse-led distraction, and combined cognitive-behavioural interventions such as hypnosis appear effective in reducing the pain and distress associated with needle procedures [15, 16]. Similarly, music therapy holds promise as an adjuvant therapy to aid the reduction of pain and anxiety in children undergoing medical procedures [17]. The successful use of these different methods is often dependent on patient preference and receptivity towards a particular therapy and the person administering the therapy.

The use of HEADPLAY PCS as a means of facilitating intravenous cannulation in a surgical setting was explored in this study. Our results suggest that its use is comparable to our current distraction techniques that employ bubbles, stickers, and verbal reassurance, in terms of anxiety, pain, and satisfaction incurred during the procedure.

This study had not been powered to detect significant differences in terms of anxiety and pain scores but satisfaction. We recognized the limitations of EMLA in reducing pain but postulated that satisfaction could still be achieved even if the cannulation was not completely pain free or the child remained slightly anxious through the use of distraction therapy with HEADPLAY PCS.

Dissatisfaction with the intravenous cannulation process in most cases was secondary to pain due to ineffectiveness of EMLA rather than the device per se; there was only one instance where the patient had low pain scores of less than 5 after intravenous cannulation but was dissatisfied because he found the device uncomfortable. EMLA is known to be rather ineffective as a topical anaesthetic. In a study involving 258 children between the ages of 5 and 18 years, EMLA was successful in 84% of venipuncture and only 51% of intravenous cannulation [18]. However, in our institution, EMLA is the only topical anaesthetic currently available to facilitate painless venipuncture and intravenous cannulation. Under the HEADPLAY PCS group, 43.3% experienced a pain score of greater than 5, while 26.7% of patients under conventional technique were noted to have the same pain score. It was rather interesting that 1 child in the conventional group and 2 children in the HEADPLAY PCS group rated their satisfaction score 100% even with relatively high pain scores of 8 and 9. In this study, we did not have a control group in which no distraction method was employed during the intravenous cannulation process. Satisfaction scores may well have been much lower if no distraction therapy had been employed at all. Cooperation with the intravenous cannulation was high in both groups with rates of 96.7% in the conventional group and 93.3% in the HEADPLAY PCS group. Cooperation was lost when pain was encountered during intravenous cannulation.

In our study, most of the children were Chinese and Malay boys with a mean age of 9 years. The fewer girls recruited in this study are a reflection of the gender disparity apparent in the study population amongst which these patients were recruited, that is, day surgery patients, the majority of whom were boys. Results from our study should therefore be interpreted in this light. We cannot conclude that there is a gender preference for this device. Children below the age of 5 years are less accepting this device. As such, our study recruited children only above the age of 5 years.

All in the HEADPLAY PCS group accepted the device that they had been introduced to prior to recruitment. Amongst them, 83% liked the device and 87% wanted to use it again if they had to undergo intravenous cannulation. When HEADPLAY PCS visors are donned, the kids are essentially blindfolded. This has the potential to allow for an immersive environment for more effective distraction but to some children, particularly those with claustrophobia, this may be a scary prospect. Hence, not every child will want to use this device.

Unlike PediSedate which also uses a headset to administer nitrous oxide in oxygen through a nosepiece used in combination with an interactive video component [19], HEADPLAY PCS cannot be modified to deliver nitrous oxide which would aid with pain reduction during invasive procedures but it may be used concomitantly for an inhalational induction if the child prefers. Unlike interactive video games which require the use of hands, HEADPLAY PCS has the advantage of allowing access to the hands for invasive procedures while providing distraction to children. Besides intravenous cannulation, HEADPLAY PCS has the potential to be applied across other minimally invasive medical procedures of short duration. While unproven, prolonged use of HEADPLAY PCS may carry the risk of developing myopia. Children who performed near work at a distance of less than 30 cm for more than 30 min continuously were noted in a study to be more likely to have myopia than those who worked at a further distance or for a shorter period of time [20]. There are other studies, however, that have not associated near work with myopia or the development of myopia [21, 22].

In this study, we could not demonstrate that HEADPLAY PCS is superior to the conventional distraction therapy that we employ during intravenous cannulation in children undergoing intravenous induction. HEADPLAY PCS is expensive. Based on our study, we cannot justify recommendations to use it routinely as part of clinical practice. However, given the diverse paediatric patient spectrum, HEADPLAY PCS may be preferred over other means of distraction therapy by some children and add to the overall satisfaction with a medical procedure that is short in duration and associated with pain that may be controlled with simple analgesics or local anaesthetics. 

## Figures and Tables

**Table 1:** Patient demographics.

Variable	Group 1: HEADPLAY (*n* = 30)	Group 2: conventional (*n* = 30)	*P* value
Age (years)	9.4 ± 2	8.77 ± 1.8	0.2
Gender (male/female)	27/3	24/6	0.28
Ethnicity (Chinese/malay/Indian)	10/18/2	14/14/2	0.558
ASA (I/II)	21/9	25/5	0.222
Previous intravenous cannula insertion (Y/N)	3/27	5/25	0.228

**Table 2:** Summary of anxiety scores.

Anxiety scores	Group 1: HEADPLAY (*n* = 30)	Group 2: conventional (*n* = 30)	*P* value
Child's self assessment score in the holding area mean ± SD (median)	3.33 ± 2.35 (4)	3.00 ± 2.7 (2.5)	0.136
Child's self assessment in the operating theatre mean ± SD (median)	4.07 ± 2.75 (4)	4.57 ± 3.58 (4.5)	0.447
MYPAS (holding area) Mean ± SD	33.1 ± 11.0	30.2 ± 10.9	0.194
MYPAS (operating theatre) Mean ± SD	37.1 ± 14.2	37.2 ± 17.7	0.796
MYPAS (iv cannulation) Mean ± SD	39.7 ± 19.5	38.2 ± 20.3	0.496
Parental assessment of child's anxiety (median)	2 (a little scared anxious)	2 (a little scared anxious)	0.554

**Table 3:** Pain and satisfaction scores.

	Group 1: HEADPLAY (30)	Group 2: conventional (30)	*P* value
Baseline pain scores	0.17 ± 0.74	0.2 ± 0.66	0.655
Pain score after iv insertion	3.77 ± 3.14	3.27 ± 3.21	0.490
Satisfaction score (mean %)	79.7 ± 26.8	76.7 ± 24.8	0.46

**Table 4:** Acceptance of HEADPLAY PCS device.

Acceptance	Frequency	Percentage
Did the child like the device?		
Like	25	83.33
Did not like	5	16.67
Did the child find the device comfortable?		
Comfortable	26	86.67
Uncomfortable	4	13.33
Would the child like to use the device the next time around if he had to undergo IV cannulation again?		
Yes	26	86.67
No	4	13.33
